# Language Analytic Ability, Print Exposure, Memory and Comprehension of Complex Syntax by Adult Native Speakers

**DOI:** 10.5334/joc.333

**Published:** 2024-01-09

**Authors:** Elodie Winckel, Ewa Dąbrowska

**Affiliations:** 1 English and American studies, Friedrich-Alexander-Universität Erlangen-Nürnberg, Philosophische Fakultät und Fachbereich Theologie, Erlangen, DE

**Keywords:** individual differences, grammar, comprehension, language aptitude, language analytic ability, memory

## Abstract

Nativist theories of language development assume that all native speakers of a particular language ultimately converge on (more or less) the same grammar, and argue that this is only possible because they are born with a genetic blueprint for language. However, a number of recent studies have found that there are, in fact, considerable individual differences in adult native speakers’ grammatical attainment. In this study, we examine some possible reasons for these differences. We examine both learner internal cognitive factors (implicit and explicit memory for sequences, non-verbal working memory, and language analytic ability) as well as an experiential factor (print exposure). In contrast to many earlier studies which focused on the temporal aspects of language processing, we are interested in the extent to which individuals are able to use grammatical cues to extract meaning from complex sentences. To minimize the effect of performance factors, sentences remained on screen while participants responded to comprehension questions (thus easing working memory load) and participants were given as much time as they needed to respond.

Our findings revealed large effects of language analytic ability and print exposure, and a much smaller effect of implicit learning. While the effect of implicit learning fits in well with current theories of language acquisition and processing, the first two findings do not. The strong relationship between print exposure and comprehension suggests that the ability to process complex syntax may depend on a particular type of language experience which is not available to all speakers. Finally, the effect of language analytic ability challenges the wide-held conviction that the ability to identify and explicitly reason about linguistic patterns is only relevant in adult second language learning.

## 1. Introduction

For many years, the general consensus in linguistics was that all native speakers converge on (more or less) the same grammar. It was, of course, acknowledged that the grammars of speakers of different dialects are somewhat different, and that speakers differ in vocabulary size and in their knowledge of some highly literary constructions (e.g. *Little did I know that…* ), but the prevailing view was that, on the whole, speakers of the same language share the same core grammar: that is to say, they have full command of the entire range of e.g. subordinate structures of their language, raising, control, etc. (see, for example Birdsong, 2004: 83; Bley-Vroman, 2009: 179; Chomsky, 1975: 11; 1986: 18; Crain, Thornton & Murasugi, 2009: 124; Dörnyei, 2009: 240; Lidz & Williams, 2009: 177; Montrul, 2008: 4; Nowak et al., 2001: 114; Seidenberg, 1997: 1600; Smith, 1999: 41; Trudgill, 1992: 130). To the lay person, this seems almost perverse: we know from everyday observation that, while all normal native speakers achieve a certain level of proficiency, they vary enormously in the extent to which they are able to produce and understand complex texts. These differences have not escaped language researchers, but for many years they had been argued away as performance issues which can be accounted for by language-independent factors such as working memory capacity (cf. Just & Carpenter, 1992; King & Just, 1991; Miyake et al., 1994; Just, Carpenter & Keller, 1996; Crain et al., 1990). In other words, the consensus was that all native speakers share the same competence, even if this is not always reflected in actual language use.

This view has been challenged in recent years by a growing number of carefully designed studies using tasks that place minimal demands on processing resources and control for linguistically irrelevant performance factors. These studies demonstrate considerable individual differences in speakers’ ability to use grammatical cues to extract meaning from utterances (for recent reviews, see Dąbrowska, 2012; 2015; Hulstijn, 2015; Kidd, Donelly & Christiansen, 2018). As a consequence, a growing number of linguists have started to recognize the “inconvenient truth” that individual differences are a pervasive feature of language acquisition and processing (Kidd et al., 2018).

With this realisation, research is increasingly shifting to the reasons for individual differences. These are interesting not only in themselves, but also because they provide important clues about the cognitive underpinnings of language acquisition and processing. In this paper, we build on this tradition and investigate the effects of individual differences in implicit and explicit memory, working memory, language analytic ability and print exposure on individual differences in the ability to comprehend complex syntactic structures. In order to investigate the latter, we developed a new, and rather demanding, syntactic comprehension test containing sentences involving long distance dependencies, multiple embedding, and the use of relatively subtle grammatical cues.

### 1.1 Implicit and Explicit Memory

Learning a language involves storing vast amounts of information: words and their grammatical properties, idioms and collocations, and a large number of grammatical constructions (some lexically specific and some more abstract). One might therefore expect individual differences in memory to be relevant for acquisition, and indeed, there is a considerable amount of research investigating the relationship between memory and linguistic attainment (see below). A large part of this research has focused on the distinction between declarative memory, or memory for facts and events (“knowledge that”), on the one hand, and procedural memory, which contains information about how to carry out sequences of operations (“knowledge how”). The declarative memory system is supported by the hippocampus and the medial temporal lobe. Since the information stored in this system is normally accessible to consciousness, the latter is often referred to as “explicit memory”. The contents of procedural memory, in contrast, are not consciously accessible but are expressed through better performance in skilled tasks (e.g. faster reaction times); this system is therefore a type of implicit memory. Neuronanatomically, procedural memory depends on the basal ganglia and the frontal premotor cortex.^1^

The prevailing view is that procedural/implicit memory supports the acquisition of grammar, while declarative/explicit memory supports lexical learning (see Hamrick et al., 2018; Ullman, 2015; 2016). However, while the latter part of this claim is uncontroversial, the relationship between implicit memory and grammar has not gone unchallenged (cf. West et al., 2017). Furthermore, although a number of studies have found a relationship between implicit learning and grammar, a recent meta-analysis (Oliveira et al., 2022) found that the average correlation across a large number of studies was close to zero (r = .04) and not significant.

Furthermore, there is some evidence to suggest that explicit memory may also be relevant for first language (L1) grammar. For example, West et al. (2017) found a significant relationship between grammatical comprehension (assessed using TROG-2) and performance on declarative memory tasks in seven- to eight-year-old English-speaking children, with correlation coefficients ranging from .25 to .52. Conti-Ramsden et al. (2015), in contrast, found a positive but non-significant relationship (r = .25, p = .096) between declarative memory and TROG in their sample of slightly older children (ages from 8;6 to 11;5). It should be noted, however, that their sample was considerably smaller (45 as opposed to 101 children for West et al.). Moreover, since the main focus of the study was to compare SLI and typically developing (TD) children, they excluded TD children whose receptive language skills were 1 SD or more below the group mean. While this move was motivated by the design of the study, it obviously resulted in less variation and could be responsible for the non-significant result.

Two other studies (Lum & Kidd, 2012 and Kidd & Kirjavainen, 2011) examined the relationship between explicit/declarative memory and children’s production of past tense verb forms in English and Finnish respectively. Lum and Kidd found no direct relationship between declarative (or procedural) memory and performance on the language task; however, they found that declarative memory predicted vocabulary, which in turn predicted morphological knowledge. Kidd and Kirjavainen found very weak, marginally significant effects of declarative memory for some classes of irregular verbs, but not for regulars, and no significant effects of procedural memory.

Finally, Llompart and Dąbrowska (2020) examined the relationship between memory and L1 grammatical proficiency (assessed using an acceptability judgement task) and memory in adult speakers. Their study revealed a robust relationship between grammar and explicit memory (r = .64); the correlation between grammar and implicit memory, in contrast, was close to zero.

Thus, the results of previous research on the relationship between grammatical abilities on the one hand and the two memory systems are mixed, with some studies reporting a relationship with implicit memory, some with explicit memory, and some failing to find any significant relationships.

### 1.2 Working Memory

While declarative and procedural memory involve the storage of knowledge for long periods of time, working memory is often seen as the mental “workspace” in which information is temporarily stored and manipulated during processing – similar to the random access memory of a computer. It seems reasonable to assume that working memory (WM) is relevant for language. During language comprehension, for instance, listeners or readers must recognize words, identify the contextually relevant meanings and use grammatical cues to integrate them into a coherent structure; working memory is thought to provide a space in which information is temporarily held while being processed. And indeed, many previous studies have demonstrated a relationship between WM capacity – traditionally assessed using complex span tasks in which participants must store and manipulate information simultaneously (cf. Conway et al., 2005) -- and performance on a variety of language comprehension and production tasks. Daneman & Merikle’s (1996) meta-analysis reports an average correlation of .52 between measures of verbal WM and “specific” language tasks (e.g. assigning pronominal reference, making inferences, detecting ambiguity) and a somewhat weaker correlation (.48) between measures of non-verbal WM (e.g. operation span, see below) and language tasks. These results appear to support the capacity theory of comprehension, which maintains that WM capacity constrains language comprehension: in other words, individuals with smaller capacity are unable to process more complex structures (Just & Carpenter, 1992; see also King & Just, 1991; Miyake et al., 1994; Just, Carpenter & Keller, 1996).

This conclusion, however, has been challenged by Caplan and Waters (1999). The authors distinguish between interpretive and post-interpretive processes, which call on different WM (sub)systems, and therefore different resource pools. According to them, interpretive processes, which include word recognition, lexical access, and syntactic analysis (including thematic role assignment) occur first. These processes are online (word by word, or even sound by sound) and obligatory. Post-interpretive processes occur later and involve accessing the meaning of the sentence in order to perform other cognitive activities (e.g. answering a comprehension question). For Caplan and Waters, what is measured by complex span tasks is only relevant for post-interpretive processes. Interpretive processes, on the other hand, are supported by a specialised WM independent of the general-purpose system.

An alternative explanation for the observed correlations between verbal working memory and language tasks is offered by constraint-based models (see e.g. MacDonald & Christiansen, 2002; MacDonald & Seidenberg, 2006), which maintain that language processing involves the rapid combination of different partially informative information sources. In this approach, linguistic knowledge is stored in a large interactive network in long term memory. This network is “co-opted” by verbal working memory tasks; thus, such tasks do not measure a separate WM capacity but “a person’s skill in encoding and maintaining verbal information” (Schwering & MacDonald, 2020: 11). Constraint-based models, therefore, predict a strong relationship between language tasks and verbal (but not non-verbal) working memory tasks.

### 1.3 Language Analytic Ability

There is ample evidence that the individual differences in grammatical skills in second language (L2) learners are partly due to differences in language aptitude, and specifically the learners’ grammatical sensitivity, as measured, for example, by the Words in Sentences subtest of the Modern Language Aptitude Test (MLAT), as well as their inductive language learning ability, which is assessed by tests such as the Language Analysis subtest of the Pimsleur Language Aptitude Battery (PLAB). Two recent meta-analyses of this research can be found in Li (2015) and Li (2016).

Grammatical sensitivity and inductive learning ability share a number of similarities. Both are most relevant for grammar learning; and the tests that measure them are strongly metalinguistic and require conscious effort; not surprisingly, performance on such tests correlates strongly with intelligence (Sasaki, 1996; Wesche et al., 1982). Therefore, these two aspects of language aptitude are sometimes grouped under the term *language analytic ability* (Li, 2016; Skehan, 2002).

It is generally assumed that language analytic abilities are only relevant for late L2 acquisition; L1 and early L2 grammatical development, in contrast, are supposed to depend (almost) entirely on implicit processes. If correct, this would imply a “fundamental difference” (Bley-Vroman, 1989; 1990) between L1 and L2 learning. However, several recent studies (e.g. Dąbrowska, 2018; Llompart & Dąbrowska, 2023; Prela et al., 2022) have found that language analytic ability is also a reliable predictor of L1 grammatical proficiency, thus challenging the fundamental difference hypothesis.

### 1.4 Print Exposure

There is considerable evidence that individual differences in grammatical knowledge and processing in adult native speakers are influenced by the amount of exposure to printed text. People who read more produce more tokens of some complex syntactic structures – such as passive relatives – which are more frequent in written texts (Montag & MacDonald, 2015). They also read faster and show different patterns of eye-fixations and word-by-word reading times, including larger wrap-up effects (Ng et al., 2020; Payne et al., 2020), engage more in predictive processing (Favier et al., 2021; Ng et al., 2018), perform slightly better on grammaticality judgement tasks (Favier and Huettig 2021) and are more likely to rely on syntactic rather than semantic cues when interpreting ambiguous pronouns (Langlois & Arnold, 2020).

More relevant for the purposes of the current study is research examining the relationship between print exposure and grammatical comprehension. Here the results are somewhat inconsistent. Dąbrowska (2019) and Street & Dąbrowska (2010) report a significant positive correlation between these variables; Acheson et al. (2008) found a small positive correlation which, however, was not significant; and Misyak and Christiansen (2012) found a significant correlation between ART and one of the three sentence sets they used; however, the relationship was not significant in the regression analysis. Finally, a training study by Wells et al. (2009) found that additional experience with written object relatives resulted in shorter reading times but had no effect on accuracy.

Stronger effects of literacy were observed in two studies which compared the grammatical abilities of adult native speakers of Spanish who are just beginning to learn to read with those of late literates (who learned to read as adults) and early literates (who learned to read as children and thus have a lifetime of reading experience behind them). The first study (Dąbrowska, Pascual & Gómez-Estern, 2022) examined the ability to comprehend a relatively difficult syntactic structure, namely object relatives, and the second (Dąbrowska, Pascual, Gómez-Estern & Llompart, 2023) the ability to provide past tense forms of novel verbs. Both studies observed very large group differences favouring the more literate participants.

The effects of literacy on grammatical comprehension can be explained in several ways. First, written texts tend to be syntactically more complex than spoken texts (Cameron-Faulkner & Noble, 2013; Karlsson, 2009; O’Donnell, 1974 – but see also Beaman, 1984). Thus, individuals who read more have more experience with complex structures, and therefore more opportunities to practise them, which could result in stronger representations and hence better performance. Secondly, when reading or writing, language users can process sentences at their own pace and backtrack or edit as necessary, which is not possible during speaking or listening. Thus, written representations can act as a kind of “processing crutch” which enables language users to understand and produce structures that they might not be able to process in the spoken modality. Once these structures have been practised in writing, their representations might become strong enough to be accessed under time pressure. In this way, reading and writing may act as “training wheels” for processing spoken language (cf. Dąbrowska, 2021). Finally, exposure to written representations could also facilitate the development of grammatical skills in more indirect ways, for example by promoting the development of metalinguistic skills and/or verbal working memory.

### 1.5 This Study

The study we present here investigates the extent to which individual differences in the ability to use grammatical information in comprehension can be explained by the five factors discussed above (implicit and explicit memory, working memory, language analytic ability and print exposure). To ensure that any observed effects are relatively specific and not simply due to familiarity with the test-taking situation or engagement with the experimental tasks, we also include two control measures which could be expected to correlate with general test taking abilities and engagement, but not with grammatical understanding. These include mental calculation (a secondary measure used in our working memory task) and a measure of ability to stay focused on a task (sustained attention).

## 2. Method

### 2.1 Participants

We recruited 79 UK participants via Prolific (https://app.prolific.co/). The participants (28 males and 51 females) were monolingual native speakers of English living in the UK. The mean age was 45.1 (range 23–73) and the participants had spent had spent on average 15.5 years in full-time education (range 10–24). Forty-three either had or were studying for a university degree (28 B.A., 12 M.A. and three Ph.D); of the remaining 36 participants, five had a Higher National Diploma, six had A levels or equivalent and five had a BTEC or equivalent; thus, the sample is relatively highly educated. Thirteen participants were unemployed, 14 had relatively low-skill jobs (e.g. healthcare worker, retail assistant, cleaning technician), and 41 held professional positions (e.g. radiographer, civil engineer, accountant). The remaining participants were retired (eight participants) or students (three participants).

All participants received 15 pounds of financial compensation for taking part in the experiment, regardless of the time they took to complete the study. On average, the study took 80 minutes to complete.

### 2.2 Tasks

*Implicit memory for sequences:* This task was based on the Visual Statistical Learning (VSL) task developed by Siegelman, Bogaerts and Frost (2017). The task tested participants’ ability to identify triplets (i.e. sequences of three complex shapes that always follow each other in the same order), without having been told that the shapes they observed were organised into triplets. The task consisted of a familiarisation phase and a test phase. In the familiarisation phase, participants were presented with a series of complex black shapes. Each shape appeared in the centre of the screen for 1000 ms. The shapes were distributed across 12 blocks, each block containing the same eight triplets, repeated twice, in a pseudo-random order. In the test phase, knowledge of the triplets was tested by means of a forced-choice task in which participants had to discriminate between familiar and foil sequences. The score reflects the proportion of correct answers in the test phase.

*Explicit memory for sequences:* Like the preceding task, this task involved a familiarisation phase in which participants were presented with triplets of (new) shapes and a test phase assessing learning. However, the instructions and the mode of presentation were different, in order to encourage explicit learning. Participants were explicitly told that the shapes came in triplets and asked to try to memorise the sequences within triplets; furthermore, a fixation cross was presented at the beginning of each triplet, thus marking triplet boundaries. There were eight triplets, each presented seven times. The score was the proportion of correct responses in the test phase.

*Sustained attention:* Sustained attention was assessed using a modified version of the Continuous Performance Task-X (CPT-X) originally developed by Rosvold et al. (1956). Participants saw letters appear one by one in the centre of the screen in rapid succession; their task was to press the space key when the letter was an X. Individual performance was assessed by means of d’ scores (Huang & Ferreira, 2020), which take into account hit rates (hit = when participants correctly identified the target letter) and false alarm rates (false alarm = when participants responded with a button press to a non-target letter).

*Language analytic ability:* Language analytic ability was measured using the Sentence Pairs task (Llompart & Dąbrowska, 2023) which is an adaptation of the Words in Sentences subtest from MLAT (Carroll & Sapon, 1959). This is a forced choice task in which participants were asked to identify words in two sentences that had the same function. In each trial, participants were presented with two sentences, as in (1):

(1) a. Bethany SANG a nice song in front of her friends.
      b. Every **Saturday, John reads** the newspaper at home while **having** a **coffee**.

Participants were then asked to choose which of the words in bold in (1b) was most similar to the word in capitals in (1a), the correct answer being “reads”. The test consists of 32 items; the score is the proportion of correct responses.

*Working memory:* Most studies of language processing use the reading or listening span task as a measure of working memory capacity. These tasks involve reading or listening to a series of sentences and answering comprehension questions about them while at the same time trying to remember the last word in each sentence for later recall. Thus, these tasks involve both a processing component and a memory component, both of which must be executed simultaneously.

Note that reading and listening span tasks require the participant to process sentences for comprehension. Since our dependent variable is also a measure of comprehension, using a verbal span task is potentially circular in that establishing a correlation between the span task and a comprehension measure would simply show that comprehension correlates with comprehension. In order to avoid this circularity, we used an operation span task (Ospan), in which participants were asked to solve simple mathematical problems while retaining a sequence of letters in memory.

Our task was based on von der Malsburg’s (2015) implementation of the Operation span task described in Conway et al. (2005). The task began with two training phases. In the first of these, participants were presented with equations (e.g. “( 6 + 5 ) × 4 = 44”) and had to press the c key if the equation was correct and the i key if the equation was incorrect. After they responded, participants were given feedback (a green tick if the response was correct and a red cross if it was incorrect). There were four practice items in this phase. In the second training phase, participants saw an equation displayed on the screen, had to press the c or i key, received feedback, and then a letter was displayed on the screen for 1000ms. After a sequence of three equations and three letters, the participants were asked to recall the letters in the correct order. This series of three operations followed by a recall probe was repeated four times.

The test phase was similar to the second training phase, except that participants no longer received feedback on the equations: they simply saw an equation on the screen and had to indicate whether it was correct. After this, a letter was displayed for 1000ms, followed by the next equation, and so on. At the end of the sequence of equations and letters, the recall screen appeared. The recall screen prompted participants to type the letters in the correct order. The expected number of letters was indicated and participants were asked to type a question mark (?) in the correct position in the sequence if they were unable to recall a particular letter. The recall screen also provided participants with information about the percentage of correct responses on the arithmetic task. Participants were told that they should be at least 85% correct on this task and were asked to monitor their performance. The purpose of this was to ensure that they did not simply focus on the recall task. We excluded the results from participants who were below a threshold of 80% correct.

The sequences of equations and letters differed in length (from three to seven equation+letter combinations). There were three sequences for each length (three sequences of three equations and three letters, three sequences of four equations and four letters, and so on), for a total of 15 sequences. The 15 sequences were presented in random order.

The score for this task is the total number of letters that participants remembered correctly and in the right position over the whole test phase. As argued by Friedman and Miyake (2005), this is a more fine-grained measure of working memory than the traditional measure, i.e. working memory span (the longest sequence recalled correctly on at least two-thirds of all trials). In addition, we also use the proportion of correct responses on the arithmetic problems as a measure of participant engagement with the task.

*Print exposure* was assessed using a computerised adaptation of the Author Recognition Test (ART; Acheson Wells & MacDonald, 2008). In this task, participants are presented with a list of 130 names. Half of these are names of well-known authors; the other half are foils. Participants are asked to indicate which names belong to authors. In our adaptation of the task, the names were presented one at a time and participants responded by clicking either a “yes” or a “no” button. Since this was an unsupervised online experiment, participants had only five seconds to make their choice -- enough time to make a decision, but too short to look up the answer. We computed d’ scores by participant, taking into account hit rates (hit = when participants answered “yes” for a real author) and false alarm rates (false alarm = when participants answered “yes” to a foil). Computing d’ scores instead of total scores leads to better reliability for the computerized version of the ART (Wright et al., in prep.).

*Grammatical comprehension task:* Our dependent variable in this experiment was participants’ ability to use syntactic cues to interpret four different types of sentences (see Table 1). Three of these (Complex NPs, X-Is-Difficult-to-Answer constructions, and Reduced Relatives) are relatively complex structures which involve two levels of embedding and long-distance dependencies:

- Sentences with Complex NPs contained a noun phrase of the form [the fact that VERBing NP is ADJ] in the subject position of an embedded clause (e.g. *Linda complained that the fact that cycling in the main square is forbidden annoys tourists*.).
- X-Is-Difficult-to-Answer constructions (cf. Herbst and Hoffmann, under review) had the form [NP1 will be ADJ to V1 NP2 to V2] (e.g. *James will be easy to persuade Walter to help*.), where the missing object of the nonfinite verb in the sentence final position (*help*) is understood to be coreferential with the subject of the main clause (*James*). Previous research (e.g. Chipere, 2001; 2003; Dąbrowska, 1997) has shown that sentences with Complex NPs and X-Is-Difficult-to-Answer constructions are difficult for many adults, so we expected considerable individual differences in performance on these items.
- The third structure were sentences containing a reduced relative clause inside another reduced relative clause inside the subject NP (e.g. *A child [staring at a dog [chasing a postman]] was afraid*.). Scholes and Willis (1987) showed that children between 8 and 10 as well as semi-literate adults exhibited very high error rates (from 35% to 60%) when interpreting such sentences. Since structures which are acquired late tend to show much more variation in adults than early-learned structures (Brooks & Sekerina, 2005; Dąbrowska, 2018; Dąbrowska, Pascual & Gómez-Estern, 2022; Indefrey, 2002), we expected this kind of item to pose difficulties for at least some adult speakers.
- The final structure were Ditransitives in which the word immediately after the verb could function either as a determiner or a pronoun (e.g. *her*) followed by two nouns and a determiner (e.g. *Mr Peters showed [her baby] [the pictures]* and *Mr Peters showed [her] [the baby pictures]*). In order to interpret such sentences, the comprehender must be able to correctly identify the constituents and their roles: in the example just given, they must determine whether the indirect object is *her* or *her baby*. The only available cue is the position of the determiner *the*. Thus, unlike the other three sentence types, the Ditransitive items were relatively simple syntactically but required the comprehender to make use of a relatively subtle grammatical cue. Scholes et al. (1976) have shown that children continue to misinterpret such sentences throughout middle and late childhood; the rationale for their inclusion was thus the same as that for the Reduced Relatives items.

**Table 1:** Example items for the grammatical comprehension task.


STRUCTURE	EXAMPLE	QUESTION (RESPONSE OPTIONS)

Complex NP	(2) Linda complained that the fact that cycling in the main square is forbidden annoys tourists.	What did Linda complain about? (That tourists are annoyed. / That cycling is forbidden in the main square.) What is forbidden? (Complaining about the cycling restrictions. / Cycling on the main square.) What annoys the tourists? (That one is not allowed to cycle in the main square. / That Linda complained about cycling restrictions.)

X-Is-Difficult-to-Answer	(3) James will be easy to persuade Walter to help.	Who might be helped? (James / Walter) What will be easy? (Persuading Walter to help James. / For James to help Walter.) Who will find it easy to do something? (Someone not mentioned in the sentence / James)

Reduced Relatives	(4) A child staring at a dog chasing a postman was afraid.	Who was afraid? (A child / A postman) Who chased someone? (A human / An animal) (**control**)

Ditransitives	(5) Mr Peters showed her baby the pictures. (6) Mr Peters showed her the baby pictures.	Who saw something? (A woman / A baby) Who showed someone something? (Mr Peters / Someone not mentioned in the sentence) (**control**)


There were eight items for each sentence type, giving a total of 32 test items. For the Ditransitives, half of the items had a structure analogous to example (5) in Table 1 and the other half were like (6).

Comprehension was assessed by means of a two-alternative forced choice task. Complex NP and X-Is-Difficult-to-Answer items had three experimental questions each and the Reduced Relatives and Ditransitives had one question. The Ditransitives and Reduced Relatives items also included control questions which probed participants’ ability to identify the subject and object respectively. In both cases, the subject or object was a simple NP which occurred in the canonical position immediately before or after the verb, so we did not expect them to pose any difficulty for adult native speakers. These questions were included to enable us to determine whether participants had engaged with the task.

To avoid fatigue, the experimental items were divided into two blocks, each block containing half of the items for each type of construction. The items within blocks and the questions for each item were presented in a pseudo-random order with the constraint that items belonging to the same condition should not occur immediately next to each other. Since we were interested in whether participants were able to extract the relevant information from the stimuli and not in how good they were in remembering them, the stimuli sentences remained on the screen while participants answered the comprehension questions.

We employed two scoring methods: proportional scores, which were computed by dividing the number of correct answers by the total number of questions for a given item (excluding control questions) and an “all-or-nothing” score where an item was considered as correct (1) if the participant answered all the experimental questions correctly and as incorrect (0) otherwise. The two scoring methods were very highly correlated (r =.96), so “all-or-nothing” scores were used in all subsequent analyses.

### 2.3 Procedure

**Figure 1:**
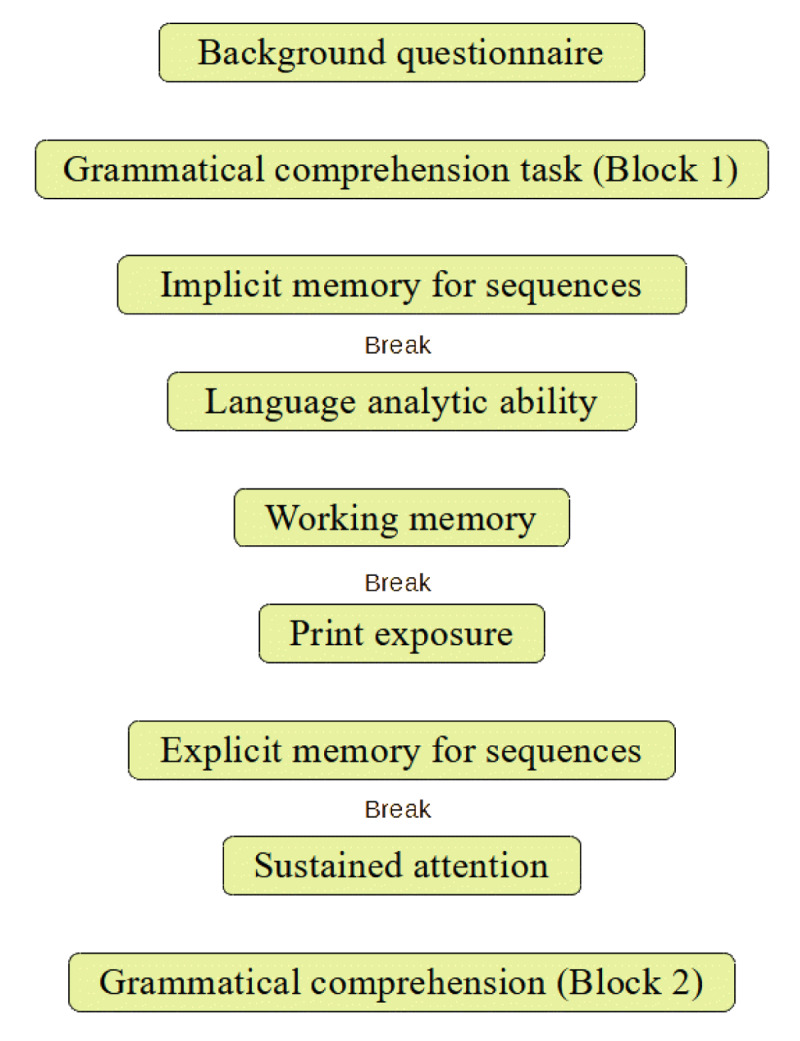
Experimental design.

Before starting the experiment, participants were invited to fill out a background questionnaire in which they were asked to provide basic demographic information (age, gender, occupation, years spent in full-time education, highest educational attainment and native language). The experiment consisted of seven tasks (one of which was divided into two blocks), with three breaks during which the participants were invited to do some guided gentle gymnastics movements. Figure 1 shows the order in which the tasks were presented to participants.

The study was conducted via the online experimental platform Gorilla (gorilla.sc; Anwyl-Irvine et al. 2020).

## 3. Results

Seven of the participants did not reach the minimum score of 80% in the calculations related to the working memory test (Ospan task). We removed the working memory data of these seven participants. The missing data were then imputed using the mice package (version 3.14.0; van Buuren & Groothuis-Oudshoorn, 2011) in R (version 4.2.1; R Core Team, 2022).

We also identified six outliers in the Author Recognition Task, who had timed out for over forty authors (between 42 and 108). Since the most likely reason for such multiple timeouts is that participants tried to cheat by looking up authors on the internet, we removed the print exposure data of these participants.

The data and R code of the project are publicly available at https://osf.io/usqme/?view_only=e884475703e74130bb9112cd5d11aaa1.

### 3.1 Descriptive Statistics

Table 2 reports the descriptive statistics and reliability estimates for all the measures in our experiment. Split-half reliability estimates were computed averaging the results of 5000 random splits using the splithalf package (version 0.8.2; Parsons, 2021) in R, except for the d’ scores, where the reliability estimate was computed by averaging the results of 1000 random splits using the splithalfr package (version 2.2.0; Pronk 2021). Cronbach’s coefficient alpha was computed using the psych package (version 2.3.9; Revelle 2023). Reliability estimates were acceptable for all tasks. They were also relatively high for individual structures tested in the grammatical comprehension task, with the exception of Ditransitives.

**Table 2:** By-participant mean value, standard deviation (SD), median value, interquartile range (IQR, with indication of the first and third quartiles), range (minimum and maximum), split-half reliability (Spearman-Brown corrected reliability estimate) and Cronbach’s coefficient alpha for all measures.


MEASURE	MEAN	SD	MEDIAN	IQR	RANGE	SPLIT-HALF RELIABILITY	CRONBACH’S ALPHA

Complex NPs	.33	.33	.25	0–.56	0–1	.85	.70

X-Is-Difficult-to-Answer	.22	.28	.12	0–.31	0–.88	.84	.59

Red. Relatives	.85	.22	1	.75–1	0–1	.78	.61

Ditransitives	.86	.15	.88	.75–1	.25–1	.42	.23

Control	.97	.05	1	.94–1	.75–1	-	-

Compr.aon	.57	.18	.56	.45–.69	.22–.97	.79	.88

Compr.RT	8256	3588	6946	5718–9927	3863–22494	.70	-

Calculation	.91	.09	.94	.89–.96	.42–1	.88	.87

WM	55.13	15.29	58	49–65	4–75	.87	.89

Print.exp	1.30	0.76	1.24	0.78–1.81	-0.11–2.87	.90	.94

Implicit	.58	.15	.57	.48–.67	.26–.95	.75	.77

Explicit	.60	.21	.56	.40–.79	.23–.98	.90	.90

LgAnalytic	.57	.23	.59	.47–.75	.03–.94	.88	.91

Attention	4.39	0.8	4.53	4.23–4.93	0.5–5.07	.76	.98


*Note*: Compr.aon = mean proportion correct (all-or-nothing score) for each item on the comprehension task; Compr.RT = comprehension reaction time (for correct and incorrect responses); Calculation = mean proportion of correct responses in the calculation part of the Ospan; WM = working memory (Operation span task); Print.exp = Print exposure (Author Recognition Task, d’), Implicit = implicit memory; Explicit = explicit memory, LgAnalytic = language analytic ability (Sentence Pairs test); Attention = Sustained Attention (d’).

As anticipated, participants were at ceiling on the control questions, indicating that they had understood the task, were cooperative etc. The scores for reduced relative clauses and Ditransitives were also relatively high, but not quite at ceiling. The other two structures (Complex NPs and X-Is-Difficult-to-Answer) were considerably more difficult. The mean overall proportion of correct responses on the grammatical comprehension task was .57, with an interquartile range from .45 to .69, indicating considerable variation in individual performance.

### 3.2 Correlational Analysis

Table 3 shows the correlations between all measures used in our study. As we can see, grammatical comprehension shows relatively robust correlations with language analytic ability and print exposure, and a much weaker correlation with implicit learning and calculation. It is worth noting that explicit and implicit sequence learning are relatively strongly correlated, which is most likely due to the fact that the two tasks were very similar. Language analytic ability is strongly correlated with print exposure and the calculation score.

**Table 3:** Correlation matrix for all measures.


	COMPR.AON	COMPR.RT	CALCULATION	WM	PRINT.EXP	IMPLICIT	EXPLICIT	LGANALYTIC	ATTENTION

Compr.aon		–.07	.26*	.22*	.58***	.26*	.21	.68***	.11

Compr.RT	–.07		.17	.01	–.20	–.05	.02	.13	.01

Calculation	.26*	.17		.22	.22	.14	.27*	.43***	.14

WM	.22*	.01	.22		.05	.16	.16	.27*	.29*

Print.exp	.58***	–.20	.22	.05		.14	.19	.45***	.07

Implicit	.26*	–.05	.14	.16	.14		.53***	.19	.20

Explicit	.21	.02	.27*	.16	.19	.53***		.30**	.36

LgAnalytic	.68***	.13	.43***	.27*	.45***	.19	.30**		.21

Attention	.11	.01	.14	.29*	.07	.20	.36**	.21	


*Note*: Correlations of .22 and above are significant at the .05 level (without correction for multiple comparisons). * p < .05. ** p <.01. *** p < .001 level.

The correlation between reaction times and accuracy comprehension task was close to 0 (r = –.07), indicating that there was no speed-accuracy tradeoff.

Table 4 shows the correlations between the different sentence types in the comprehension task. While all the correlations are positive, they range from moderately strong to very weak. It is noteworthy that the correlation between the two most difficult structures, namely X-Is-Difficult-to-Answer constructions and Complex NPs is the highest. The relatively low correlations between the easier structures are most likely due to the fact that performance on these two structures is close to ceiling (see above).

**Table 4:** Correlations between structures on the comprehension task (all-or-nothing scores).


	COMPLEX NPS	X-IS-DIFFICULT-TO-ANSWER	RED. RELATIVES	DITRANSITIVES

Complex NPs		.64	.38	.07

X-Is-Difficult-to-Answer	.64		.30	.18

Red. Relatives	.38	.30		.37

Ditransitives	.07	.18	.37	


### 3.3 Regression Analyses

To examine which factors contribute to differences in comprehension, we fitted a mixed-effects model with a logistic linking function using the lmerTest package (version 3.1-3; Kuznetsova et al., 2017) in R. The data and the R code used in the analysis are provided in the supplementary materials. The dependent variable was the all-or-nothing score for each item in the comprehension task.^2^ We included the following predictors: working memory (measured by the score on the Ospan task), print exposure (measured by the d’ score on the ART), implicit memory for sequences, explicit memory for sequences, language analytic ability and sustained attention. We added random intercepts for participants and items.^3^

The model (summarized in Table 5) shows three significant main effects: language analytic ability, print exposure and implicit learning. These factors have positive coefficients, indicating that higher scores for these abilities are associated with better performance on the grammatical comprehension task. The estimate for language analytic ability is the highest, indicating a somewhat larger effect, while the estimate for implicit memory is the lowest, indicating that this effect is smaller than the other two.

**Table 5:** Fixed effects for the model predicting comprehension scores.


	ESTIMATE	STANDARD ERROR	Z VALUE	*p* VALUE

Intercept	0.6482	.4271	1.518	.1291

LgAnalytic	0.8735	.1528	5.715	1.10 × 10^-8^

Print.exp	0.5469	.1384	3.953	7.73 × 10^-5^

Implicit	0.2854	.1434	1.990	.0466

Calculation	–0.1018	.1326	–0.768	.4425

Explicit	–0.1126	.1508	–0.746	.4555

WM	0.0940	.1304	0.721	.4710

Attention	–0.0851	.1294	–0.658	.5105


All other predictors (i.e. calculation, explicit memory, working memory and attention) had lower estimates. They were not significant predictors in our model. Even though many predictors correlate with each other, no multicollinearity could be detected.

We also attempted to fit a model with sentence type and its interactions with the other predictors to determine if they affect performance on all constructions in the same way, but it did not converge. We therefore ran four different models, one for each sentence type. Appendix 7.3 provides summaries of these models. Language analytic ability was a significant predictor for all sentence types. Print exposure was a significant predictor for X-Is-difficult-to-Answer and Ditransitives, but only marginally significant for the other sentence types. Implicit memory was not significant in any of these four additional models, which could be due to of a lack of power. Surprisingly, working memory was a significant predictor for Ditransitives. Since this was the syntactically simplest construction in the test, this could be a type 1 error.

## 4. Discussion

### 4.1 Language Analytic Ability

Our results revealed a robust effect of language analytic ability. This finding is in line with recent studies by Dąbrowska (2018), Prela et al. (2022) and Llompart and Dąbrowska (2023), which also found this aspect of “foreign” language aptitude to be a significant predictor of performance on tasks tapping grammatical knowledge in adult native speakers. It should be noted that these earlier studies used a variety of measures of aptitude and grammatical ability. Thus, Dąbrowska (2018) assessed aptitude using the Language Analysis subtest of the PLAB, while Prela et al. and Llompart and Dąbrowska’s study 2 employed the Sentence Pairs task used here; and Llompart and Dąbrowska’s study 1 employed both measures. Grammatical proficiency was assessed by means of picture selection in Dąbrowska (2018) and Llompart & Dąbrowska’s study 2 and by means of a grammaticality judgement task in their study 2 and in Prela et al. (2022). Dąbrowska (2018) and the two studies in Llompart and Dąbrowska (2023) report remarkably similar correlations between performance on the aptitude test and the grammar task: .46 in Dąbrowska’s study; .48 and .45 for Sentence Pairs and Language Analysis respectively in Llompart and Dąbrowska study 1; and .43 in Llompart & Dąbrowska’s second study. The correlation reported by Prela et al. is somewhat lower (.37), which is most likely due to ceiling effects (the participants were 93% accurate on the native language grammar task). In the present study the correlation was higher (.68); this is most likely because our measure of grammatical proficiency was considerably more difficult than those used in the previous studies, and hence the differences in performance between speakers were much larger. The fact that all these studies obtained very similar results in spite of the fact that they used different measures provides strong convergent evidence that the relationship we report here is real.

It is also worth noting that all the correlations between language analytic ability and grammatical knowledge observed in these five studies are all higher than the correlations typically reported in L2 studies: the average correlation coefficient in Li’s (2015) meta-analysis of L2 research of the effects of language analytic ability on grammatical proficiency is .31 (95% CI = .25–.36). Language aptitude tests such as the MLAT and PLAB were originally developed to predict foreign language achievement in instructional contexts, particularly in the early stages of language learning. However, they have also proven to be good predictors of second language learning in naturalistic settings, even at later stages. In fact, some L2 researchers (e.g. McLaughlin, 1990; DeKeyser, 2000) have proposed that the relationship between aptitude and achievement may be even stronger in naturalistic contexts, because naturalistic learners have to discover the rules of the language for themselves, without the help of a teacher. The results reported here and the earlier studies we reviewed suggest that this may also be true of L1 learners.

### 4.2 Print Exposure

While there is considerable evidence that print exposure is related to vocabulary size and reading comprehension (for a meta-analysis see Mol & Bus, 2011), relatively few studies have examined the relationship between print exposure and grammatical comprehension in adults, and, as noted in the introduction, they have produced somewhat inconsistent results. The effect observed in the current study (r = .55) is larger than that observed in most previous studies. This could be due to the fact that our grammatical comprehension task was more demanding than those used in previous research.

### 4.3 Implicit Memory for Sequences

As noted in the introduction, it is widely assumed that the acquisition of native language grammar depends largely on implicit learning abilities, and a number of studies report significant correlations between procedural/implicit learning abilities and grammatical abilities. However, not all studies found such an effect; and in fact, a recent meta-analysis (Oliveira et al., 2022) found no significant relationship between these two variables. It should be noted that Oliveira et al.’s meta-analysis only included studies which used the serial reaction time task as a measure of implicit learning. As the authors note, such tasks are notoriously unreliable, which could explain the lack of a relationship.

The visual statistical learning task used in the current study has much better reliability (.83 in the original study by Siegelman et al., and .75 according to our estimates). With this improved measure, we did find a significant relationship. It should be noted, however, that the observed effect was rather small (r = .26, see Table 3).

### 4.4 Working Memory

As discussed above, many studies have demonstrated strong links between working memory and language comprehension. However, it is not clear whether this relationship is due to the fact that working memory capacity imposes constraints on individuals’ ability to process language (as argued for example by Just & Carpenter, 1992), whether these effects appear at the post-interpretive stage (a hypothesis put forward by Caplan & Waters, 1999), or whether verbal working memory tasks effectively measure linguistic knowledge (as proposed by Schwering and MacDonald, 2020).

Our grammatical comprehension task differed from the tasks used in most previous studies in that it alleviated the demands on working memory by leaving the sentence on the screen while participants answered the comprehension question and by allowing participants to process the stimulus sentences at their own pace (i.e. without time constraints). Under these conditions, we observed no significant effect of individual differences in operation span (with the possible exception of the Ditransitive condition), despite the fact that the sentences were very difficult, as evidenced by high error rates.

These findings seem to argue against Just and Carpenter’s capacity theory of comprehension and are consistent with Caplan and Waters’ theory. They could also be accommodated in constraint-based theories of comprehension, which predict a strong relationship between comprehension and verbal working memory tasks, but not necessarily between language comprehension and operation span tasks.

### 4.5 Explicit Memory for Sequences, Sustained Attention and Mental Calculation

None of the other measures (sustained attention, mental calculation or explicit memory for sequences) were significant predictors of grammatical comprehension.

The lack of effect for explicit memory contrasts with the findings of Llompart and Dąbrowska (2020), who observed robust correlations between performance on a grammaticality judgement task and two explicit memory measures (digit span and paired associates) in adult native speakers. This could be due to the fact that the explicit memory task used in this study was entirely non-verbal – although it should be noted that we *did* find significant effects of implicit memory, which was also assessed using a non-verbal task.

Be that as it may, the lack of any effect of explicit memory for sequences, selective attention or accuracy on the computational part of the WM task indicates that individual differences in scores on the comprehension task cannot be explained by appealing to factors such as engagement with the task, familiarity with testing, or other linguistically irrelevant performance factors: in other words, they reflect genuine differences in language knowledge.

### 4.6 Directionality of Causation

Our main interest in this study is in the reasons for individual differences in native speakers’ grammatical knowledge. Obviously, since our data is correlational, we cannot make strong inferences about causation. It is, nevertheless, useful to reflect on the plausibility of various causation scenarios involving our variables. Given a correlation between two different abilities (A and B), four scenarios are possible:

- A causes B;
- B causes A;
- reciprocal causation (A affects performance on B which in turn affects performance on A and so on); and
- the correlation is due to a third variable, C, which causes both A and B.

In this section, we discuss the plausibility of these four scenarios with regard to the three variables which emerged as significant predictors of performance on the grammar task, namely implicit learning, print exposure and language aptitude.

#### 4.6.1 Implicit learning

It is generally assumed that individual differences in implicit memory play a causal role in language acquisition. This fits in with the prevailing view of child language acquisition: children acquire grammar at a very young age (by about 4 or 6), converge on the same system in spite of large individual differences in intelligence and explicit learning ability, and the resulting knowledge is inaccessible to consciousness (in the sense that native speakers without linguistic training are generally unable to verbalise the rules of the grammar of their language). We do not necessarily agree with the premises of this argument: grammatical development is not complete by age 4 or 6 (cf. Frizelle et al., 2018; Kaplan & Berman, 2015; Nippold, 2016; Wassenberg et al., 2008), and learners do not converge on the same system (Dąbrowska, 2012; 2015). Furthermore, implicit knowledge can be a result of explicit learning, as when a skill is automatized (Kihlstrom, 1987). However, it is difficult to see how learning grammar could lead to improvement on non-linguistic tasks assessing implicit memory, or what mental ability could possibly underlie performance on a task implicit learning of visually presented shapes and grammatical comprehension (it could not be a general characteristic like intelligence, since performance on implicit learning tasks is not related to individual differences in intelligence). Therefore, the most plausible conclusion is that, other things being equal, people who are better at implicit learning acquire better command of the grammar of their native language.

#### 4.6.2 Print exposure

With regard to print exposure, it is not difficult to think of plausible scenarios in which more experience with written language would result in better command of complex grammatical structures. As pointed out in the introduction, written texts tend to be syntactically more complex. Therefore, speakers who read more are exposed to more exemplars of complex constructions, and hence have more opportunities to learn them. Alternatively, the availability of written representations may facilitate the acquisition of complex syntax by easing working memory load and allowing learners to process language at their own speed. A third possibility is that print exposure facilitates grammatical development more indirectly, for instance by promoting the development of metalinguistic skills (although this should be most relevant in earlier stages of literacy acquisition, before reading becomes fully automatic, rather than in adults) or working memory (which again seems implausible, given that working memory was not a significant predictor of performance in our study).

The opposite scenario – that knowledge of grammar has a causal effect on print exposure – might appear far-fetched at first glance; however, it is certainly not impossible. There is evidence that better grammar skills lead to better reading comprehension (Hjetland et al., 2019; Kim, 2015); and better comprehension could result in more enjoyment and hence more reading. It has been argued that such effects hold for vocabulary. In this case, the effects are almost certainly reciprocal: children with larger vocabularies find reading easier and more enjoyable, and therefore they read more; increased print exposure results in vocabulary gains which make reading even more enjoyable, and so on (cf. Mol & Bus, 2011; Stanovich, 1986; Sullivan & Brown, 2015). Such reciprocal effects are also very likely for grammar.

The final scenario – that of a third variable which affects both print exposure and grammar – is also possible. Here the most likely candidates would be parental education and socioeconomic status (SES) as well as an individual’s own education and SES, all of which are known to be good predictors of linguistic skills. However, the effects of these variables are likely to be mostly mediated by print exposure: children of educated parents are more likely to be read to in the preschool years, and encouraged to read once they have become literate themselves, than those born into low SES families; educated people are likely to have read more at school and at university than less educated individuals; people with more disposable income are likely to own more books, and therefore read more, than low-income adults, and so on. While factors related to print exposure do not account for all SES-related differences in language abilities, they do appear to account for the lion’s share of these abilities, at least for vocabulary (Sullivan & Brown, 2015).

#### 4.6.3 Language analytic ability

A number of studies have shown robust relationships between language aptitude and foreign language achievement (see Li, 2015; 2016), and it is generally assumed that the relationship is causal: that is to say, high-aptitude learners are more successful *because* the cognitive abilities assessed by aptitude tests play an important role in adult second learning. Given that the relationship between aptitude and achievement appears to be even stronger for the native language (at least with regard to grammatical sensitivity and grammatical comprehension), the question arises whether language aptitude could also play a role in first language acquisition.

**Figure 2:**
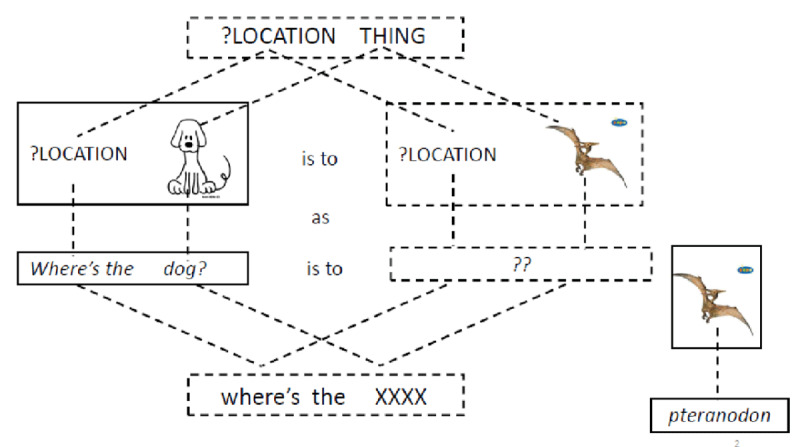
Generalisation as a proportional analogy problem. *Note*: CAPITALS and pictures represent semantic structure while lower case letters represent phonological forms.

It is not difficult to envisage how language aptitude (and grammatical sensitivity in particular) might help with learning grammar. In order to be able to produce and understand novel sentences, speakers need to generalise beyond the sentences they have previously encountered. According to usage-based models, generalisation involves analogical reasoning (Behrens, 2017; Goldwater, 2017; Tomasello, 2003). This, we argue, is true for L1 as well as L2 acquisition. Consider a learner who wants to ask about the location of a pteranodon but does not have a constructional template for location questions or a lexically specific unit which corresponds to their semantic intention. However, our learner has acquired the lexically specific unit *Where’s the dog?* as a means of asking about the location of a dog and knows the noun *pteranodon*. To produce the target question, the learner needs to recognize the overall similarity between the meaning of the lexically specific unit and their semantic intention (they are both questions about the location of an object), perform an analogical mapping between the relevant parts (the ?LOCATION substructure in their semantic intention corresponds to ?LOCATION in the semantic representation of the familiar expression; PTERANODON corresponds to DOG) and, finally, match these semantic substructures with the appropriate phonological forms (*where* corresponds to ?LOCATION and PTERANODON corresponds to *pteranodon*).^4^ In other words, the learner must in effect solve a proportional analogy problem, as illustrated in Figure 2.

However, generalisation by analogy is not without dangers (e.g. Chomsky, 1966; Searchinger, 1994). To solve the proportional analogy problem correctly, the learner needs to understand which chunk of meaning corresponds to which chunk of form and identify structural parts which play the same role in different utterances – and this is exactly what the Sentence Pairs task measures. One could even speculate that the relationship between language analytic abilities and grammar is even stronger for L1 than for L2 because L1 learners, in contrast to most adults learning a second language, have to discover the rules of grammar for themselves, without the help of a teacher or a textbook.

Thus, a causal link from grammatical sensitivity to grammatical knowledge makes sense theoretically if we assume a usage-based account of acquisition. A causal link in the opposite direction, in contrast, is implausible, given that once grammatical knowledge becomes automatized, it is not accessible to consciousness and hence presumably cannot influence performance on a highly metalinguistic task such as Sentence Pairs (see Llompart & Dąbrowska, 2023 for further discussion).

This leaves the final scenario, namely, that individual differences in performance on grammatical sensitivity as well as grammatical comprehension tasks are caused by a third, unobserved variable. The obvious candidate for this third variable would be general intelligence, which is known to correlate strongly with aptitude (Li, 2016). This is not surprising, as there is overlap in the tasks used in standard IQ and language aptitude tests (for example, both often include vocabulary and memory measures). In fact, some researchers appear to equate language aptitude with (verbal) intelligence: for instance, DeKeyser et al. (2010) assessed aptitude using a test designed to select candidates suitable for university study which contained tasks traditionally included in IQ tests (e.g. definitions, analogies and verbal reasoning).

There are, however, reasons for thinking language aptitude and IQ are distinct, though related, concepts. For second language learners, aptitude appears to be a better predictor of language achievement than intelligence (Gardner & Lambert, 1965; Li, 2019). Furthermore, the findings reported by Dąbrowska (2018) suggest that both variables contribute independently to individual differences in native speakers.

Because of the partial overlap between language aptitude and intelligence, it is difficult to distinguish between the direct causation and third variable scenarios. Perhaps the most plausible conclusion is that language analytic ability is something that emerges when general intelligence is applied to language data, or possibly to written linguistic representations. The latter formulation would explain the links that many researchers have observed between literacy and meta-linguistic awareness (Karanth et al., 1995; Nagy & Anderson, 1995; Nunes et al., 2006).

## 5. Conclusion

Our results add to the growing body of research demonstrating substantial individual differences in native language grammatical proficiency. The existence of individual differences in grammatical knowledge has important theoretical and methodological implications for language research.

One of the most powerful arguments in favour of the universal grammar hypothesis is the assumption that all native speakers of a language converge on (more or less) the same grammar. Since different learners are exposed to different input, the only way to explain this convergence, so the argument goes, is to assume a substantial body of innate knowledge. Our results, and those of other studies demonstrating the existence of substantial individual differences, appear to undermine this argument: they revealed vast individual differences in performance on all of the constructions we tested. For three of the constructions (Complex NP, X-Is-Difficult-to-Answer, and Reduced Relatives), individual scores ranged from 0% to 100%; for Ditransitives, they ranged from 25% to 100%. In contrast, participants were at ceiling (97% correct on average) on the control questions, indicating that they had understood the task, were cooperative, etc.

This raises the question whether the observed differences are indicative of differences in linguistic knowledge, or whether they are better seen as linguistically irrelevant performance factors. It is important to note in this connection that our participants were tested under ideal conditions: they were given as much time as they needed to respond, and the sentences remained on screen while they responded, thus easing working memory load. Furthermore, we observed no effect of working memory (except possibly in the Ditransitive condition). The fact that some participants performed at chance under such conditions, we would argue, suggests that they indeed lacked the relevant linguistic knowledge. Of course, it cannot be ruled out that our participants’ competence was masked by some other performance factor that we have not considered: but, we submit, the burden of proof rests with those who suggest that this is the case.

A related issue has to do with whether our results generalize to other constructions. As explained in the Method section, we deliberately chose structures which we expected to be difficult even for adult native speakers. While the sentences included in the Reduced Relative and Ditransitive conditions were relatively run-of-the-mill, our stimuli also included two admittedly unusual constructions, namely, sentences with Complex NPs and instances of the X-Is-Difficult-to-Answer construction. We observe in this connection that these sentences were modelled on examples from articles published in 1992 and 1993 in *Linguistic Inquiry* arguing for the then-current version of generative grammar, and thus presumably exemplify the types of structures generated by the rules that make up the adult native speaker’s competence (cf. Dąbrowska, 1997). It is also important to note that our participants were, on the whole, highly educated (mean 15.5 years in full time education). Based on previous research, we would expect research participants to do considerably better than individuals with less schooling – and indeed, many of the studies mentioned in the introduction observed substantial individual differences on much simpler constructions.

Our findings also challenge another widely-held assumption, namely the claim that the acquisition and processing of L1 grammar rely (almost) entirely on implicit learning. While implicit memory was a significant predictor of performance on the grammatical comprehension task, its effects were much smaller than those of language analytic ability. This suggests that conscious controlled processes also play a role, at least when it comes to relatively complex structures such as those studied here.

Finally, our findings indicate that print exposure is also a strong predictor of grammatical comprehension. Further research is needed to determine whether this effect is due to increased exposure to complex syntax in more literate participants, to the processing advantages that the written medium offers to highly skilled readers, or to more indirect effects of literacy such as metalinguistic awareness.

It is important to note that the results reported above are subject to some limitations. First, we only had one task assessing each of our predictor variables. This means that there will be task-specific limitations that may affect the degree to which the results can be generalised. In addition, the comprehension task contains only four sentence types, which are certainly not representative of all aspects of syntax. Although more tasks per predictor and more sentence types would have provided more generalizable results, we wanted to keep the study short in order to ensure that the lower literacy participants remained engaged. Another limitation of this study is the relatively small sample size, given the number of predictors. Thus, further research will be necessary to provide definitive answers to the questions raised in this study.

## Data Accessibility Statements

The data and R code of the project are publicly available via the following link: https://osf.io/usqme/?view_only=e884475703e74130bb9112cd5d11aaa1.

The de-anonymized link will be provided for the final print version.

## Additional File

An additional file for this article can be found at the following url:

10.5334/joc.333.s1 Appendix. Materials for the grammatical comprehension task, additional correlation matrix and additional regression models for each sentence type.

## Footnotes

The terms “implicit” and “explicit” on the one hand and “declarative” and “procedural” on the other are not strictly synonymous: the first distinction hinges on whether or not learning or the acquired knowledge is conscious, while the second depends on the brain system involved. In this paper, we will use a task which involves conscious and intentional learning and a task which involves incidental learning without awareness (see Method). Since we cannot be sure which brain systems are involved, we use the terms “implicit” and “explicit” in the following discussion.

We conducted an Item Response analysis to assess the unidimentionality of the items in the comprehension task with the package mirt (Chalmers 2012) in R. The Comparative Fit Index is .92, which is just below the most conservative standard of .95. Other tests for unidimentionality led to similar results: the unidimentionality is only marginal. The details are provided in the supplementary materials. Table 2 shows weak realiability for the sentence types taken on their own, but a good reliability for the comprehension task overall. The model reported here therefore uses overall comprehension scores. One possible option would have been to exclude Ditransitives due to their low reliability and the ceiling effects we could observe above. An alternative regression model without the Ditransitives gave similar results to those reported here.

Since there is only one value per participant for the cognitive measures and print exposure, random intercept for participant is the only choice. We did not include random slopes for items because adding them resulted in convergence problems. However, we selected the highest degree of complexity for which the model converged, with random slope for item by calculation score. The interested reader is referred to the R code, in which the model selection for the random variable is depicted.

Note that the learner would be able to do this even if they have not yet been able to map *‘s* and *the* onto appropriate semantic substructures: the forms *‘s* and *the* could function as placeholders for meanings that are still to be acquired.
