# Supplementary material for: Language Analytic Ability, Print Exposure, Memory and Comprehension of Complex Syntax by Adult Native Speakers
Source: J Cogn. 2024 Jan 9;7(1):7. doi: 10.5334/joc.333 (PMC10785956; doi:10.5334/joc.333)
Supplement: Appendix. — Materials for the grammatical comprehension task, additional correlation matrix and additional regression models for each sentence type. [file joc-7-1-333-s1.pdf]

7. Appendix

7.1 Material of the grammatical comprehension task

Complex NPs

(1) Tom thinks that the fact that keeping clothes clean is absolutely necessary surprises the butcher.

What does Tom think?

That the butcher is surprised.

That keeping clothes clean is necessary.

What is necessary?

That Tom thinks about keeping his clothes clean.

Keeping clothes clean.

What surprised the butcher?

That keeping clothes clean is necessary.

That Tom thinks that keeping clothes clean is necessary.

(2) Phil believes that the fact that flying planes low is very dangerous surprises the pilot.

What does Phil believe?

That the pilot is surprised.

That flying planes low is dangerous.

What is dangerous?

Surprising the pilot.

Flying planes low.

What surprises the pilot?

That it is dangerous to fly planes low.

That Phil believes that flying planes low is dangerous.

(3) Sally reported that the fact that paying rent late was not tolerated worried the student.

What did Sally report?

That the student was worried.

That paying rent late was not tolerated.

What was not tolerated?

Sally reporting about late payments.

Paying rent late.

What worries the student?

That there is no tolerance for late rent payments.

That Sally reported the late payments.

(4) Linda complained that the fact that cycling in the main square is forbidden annoys tourists.

What did Linda complain about?

That tourists are annoyed.

That cycling is forbidden in the main square.

What is forbidden?

Complaining about the cycling restrictions.

Cycling on the main square.

What annoys the tourists?

That one is not allowed to cycle in the main square.

That Linda complained about cycling restrictions.

(5) Tom thinks that the fact that keeping offices tidy is essential surprises the manager.

What does Tom think?

That the manager is surprised.

That keeping offices tidy is essential.

What is essential?

That Tom thinks about keeping offices tidy.

Keeping offices tidy.

What surprises the manager?

That it is essential to keep offices tidy.

That Tom thinks that keeping offices tidy is essential.

(6) Harry claims that the fact that changing trains often is annoying bothers the inspector.

What does Harry claim?

That the inspector is bothered.

That changing trains often is annoying.

What is annoying?

That Harry bothers the inspector.

Changing trains often.

What bothers the inspector?

That it is annoying to change trains often.

That Harry claims that changing trains often is annoying.

(7) Alistair says that the fact that using paper carelessly is wasteful surprises the pupils.

What does Alistair say?

That the pupils are surprised.

That using paper carelessly is wasteful.

What is wasteful?

Talking to pupils about saving paper.

Using paper carelessly.

What surprises the pupils?

That it is wasteful to use paper carelessly.

That Alistair says that using paper carelessly is wasteful.

(8) Kim says that the fact that raising children well is important inspires the couple.

What does Kim say?

That the couple are inspired.

That raising children well is very important.

What is important?

That the couple is inspired.

Raising children well.

What inspires the couple?

That it is important to raise children well.

That Kim says that raising children well is important.

X-Is-Difficult-to-Answer

(1) Alison will be hard to get Tim to give a loan to.

Who might give a loan to someone? [agent-active // patient-passive]

Alison

Tim

What will be hard? [action]

Getting Tim to do something.

Getting Alison to do something.

Who will find it hard to do something? [experiencer]

Someone not mentioned in the sentence

Alison

(2) Sarah will be easy to get Tim to vote for.

Who might vote for someone?

Sarah

Tim

What will be easy?

Getting Tim to vote for Sarah.

For Sarah to vote for someone.

Who will find it easy to do something?

Someone not mentioned in the sentence

Sarah

(3) Mary will be difficult to persuade Andrew to apologize to.

Who might apologize?

Mary

Andrew

What will be difficult?

Persuading Andrew to apologize.

For Mary to apologize.

Who will find it difficult to do something?

Someone not mentioned in the sentence

Mary

(4) Charles will be embarrassing to ask Willie to pray for.

Who might pray?

Charles

Willie

What will be embarrassing?

Asking Willie to pray.

For Charles to pray.

Who will find it embarrassing to do something?

Someone not mentioned in the sentence

Charles

(5) Jill will be hard to convince Lois to argue against.

Who might be argued against?

Jill

Lois

What will be difficult?

Convincing Lois to argue against Jill.

For Jill to argue against Lois.

Who will find it difficult to do something?

Someone not mentioned in the sentence

Jill

(6) James will be easy to persuade Walter to help.

Who might be helped?

James

Walter

What will be easy?

Persuading Walter to help James.

For James to help Walter.

Who will find it easy to do something?

Someone not mentioned in the sentence

James

(7) Brian will be difficult to ask Joshua to look after.

Who might be looked after?

Brian

Joshua

What will be difficult?

Asking Joshua to look after Brian.

For Brian to look after Joshua.

Who will find it difficult to do something?

Someone not mentioned in the sentence

Joshua

(8) Louise will be embarrassing to ask Ryan to support.

Who might be supported?

Louise

Ryan

What will be embarrassing?

Asking Ryan to do something.

Being supported.

Who will find it embarrassing to do something?

Someone not mentioned in the sentence

Ryan

Reduced Relatives

(1) A salesman watching a mother reading to her son was clapping his hands.

Who was clapping his hands? [narrow]

A salesman

The son

Who was reading? [control]

The mother

The son

(2) A little girl looking at a schoolmate tripping a lady in the street was very upset.

Who was upset?

A little girl

A lady

Who tripped someone?

The little girl's schoolmate

Someone not mentioned in the sentence

(3) A woman pointing at a kid hitting another kid was yelling.

Who was yelling?

A grown-up

A child

Who hit someone?

A grown-up

A child

(4) A child staring at a dog chasing a postman was afraid.

Who was afraid?

A child

A postman

Who chased someone?

A human

An animal

(5) A policeman running after a tall guy holding a baby screamed.

Who screamed?

A policeman

A baby

Who was holding someone?

A man

A baby

(6) A girl yelling at her older brother hassling a dog started to run.

Who ran?

A human

An animal

Who did the hassling?

A boy

A girl

(7) A baby smiling at a boy painting an old man on a bench stood up carefully.

Who stood up?

An adult

A baby

Who was painting?

A boy

Someone not mentioned in the sentence

(8) A student watching a thief shoving an old woman called for help.

Who called for help?

A student

An old woman

Who shoved someone?

The thief

The student

Ditransitives

(1) a. Mr Peters showed her baby the pictures.

b. Peter showed her the baby pictures.

Who saw something? [det // pronoun]

A woman

A baby

Who showed someone something? [control]

Mr Peters

Someone not mentioned in the sentence.

(2) a. The young man showed her bird the seed.

b. John showed her the bird seed.

Who saw something?

A woman

A bird

Who showed someone something?

A man

Someone not mentioned in the sentence.

(3) a. The fashion designer showed her boys the hats.

b. The fashion designer showed her the boys' hats.

Who saw something?

Some boys

A woman

Who showed someone something?

A fashion designer

Someone not mentioned in the sentence.

(4) a. Alice showed her boy's horse the shoes.

b. Alice showed her boys the horseshoes.

Who saw something?

Some boys

A horse

Who showed someone something?

Alice

Someone not mentioned in the sentence.

(5) a. Ken showed his friend's lion the tracks.

b. Ken showed his friends the lion tracks.

Who saw something?

Ken's friends

A lion

Who showed someone something?

Ken

Someone not mentioned in the sentence

7.2 Correlation of each sentence type with measures of individual differences

Table 6

Pearson correlation coefficient between by-participant all-or-nothing score on the comprehension task and other measures of individual differences in the study

	Complex NPs	Ditransitives	Red. Relatives	X-Is-Difficult-to-Answer
age	-.005	.077	.148	.076
Calculation	.138	.055	.223	.058

WM	.115	.139	.116	.142
Print.exp	.287	.176	.258	.322
Compr.RT	.061	.069	.020	-.031
LgAnalytic	.378	.180	.325	.318
Attention	.131	.011	.004	.026
Implicit	.147	.080	.115	.110
Explicit	.154	-.003	.136	.074

Note: Compr.RT = comprehension reaction time (for correct and incorrect responses); Calculation = mean proportion of correct responses in the calculation part of the Ospan; WM = working memory (Operation span task); Print.exp = Print exposure (Author Recognition Task, d'), Implicit = implicit memory; Explicit = explicit memory, LgAnalytic = language analytic ability (Sentence Pairs test); Attention = Sustained Attention (d')

7.3 Sentence types: Regression models for individual constructions

Table 7

Fixed effects for the model predicting comprehension scores for X-Is-Difficult-to-Answer

	Estimate	Standard Error	Z value	*p* value
Intercept	-2.3936	.3576	-6.693	2.18×10^{-11}
LgAnalytic	1.2341	.3455	3.572	.0004
Print.exp	0.8975	.2799	3.207	.0013
Implicit	0.2576	.2897	0.889	.3739
Calculation	-0.4422	.2897	-1.527	.1269
Explicit	-0.3917	.2956	-1.325	.1851
WM	0.4794	.2870	1.671	.0948
Attention	-0.3872	.2512	-1.542	.1232

Table 8

Fixed effects for the model predicting comprehension scores for Reduced Relatives

	Estimate	Standard Error	Z value	*p* value

Intercept	2.7558	.3405	8.093	5.81×10^{-16}
LgAnalytic	0.9380	.2874	3.263	.0011
Print.exp	0.4865	.2825	1.722	.0851
Implicit	0.3621	.2918	1.241	.2146
Calculation	0.2605	.2196	1.186	.2355
Explicit	0.0647	.3005	0.215	.8296
WM	-0.1358	.2375	-0.572	.5675
Attention	-0.2453	.2583	-0.950	.3423

Table 9

Fixed effects for the model predicting comprehension scores for Complex NPs

	Estimate	Standard Error	Z value	*p* value
Intercept	-1.4365	.4316	-3.328	.0009
LgAnalytic	1.1820	.3495	3.382	.0007
Print.exp	0.5824	.3055	1.907	.0566
Implicit	0.3457	.3213	1.076	.2819
Calculation	-0.2216	.3102	-0.714	.4750
Explicit	-0.0081	.3335	-0.024	.9807
WM	-0.2209	.2873	-0.769	.4420
Attention	-0.2452	.3084	-0.795	.4267

Table 10

Fixed effects for the model predicting comprehension scores for Ditransitives

	Estimate	Standard Error	Z value	*p* value
Intercept	2.6093	.4577	5.701	1.19×10^{-8}
LgAnalytic	0.4784	.1747	2.738	.0062

Print.exp	0.5491	.1818	3.020	.0025
Implicit	0.2547	.1745	1.460	.1444
Calculation	-0.1454	.1958	-0.743	.4577
Explicit	-0.1977	.1943	-1.017	.3090
WM	0.4030	.1425	2.827	.0047
Attention	-0.0919	.1513	-0.607	.5437
